# ERG deregulation induces IGF-1R expression in prostate cancer cells and affects sensitivity to anti-IGF-1R agents

**DOI:** 10.18632/oncotarget.3425

**Published:** 2015-03-27

**Authors:** Caterina Mancarella, Irene Casanova-Salas, Ana Calatrava, Selena Ventura, Cecilia Garofalo, José Rubio-Briones, Vera Magistroni, Maria Cristina Manara, José Antonio López-Guerrero, Katia Scotlandi

**Affiliations:** ^1^ CRS Development of Biomolecular Therapies, Experimental Oncology Lab, Rizzoli Orthopaedic Institute, Bologna, Italy; ^2^ Laboratory of Molecular Biology, Fundación Instituto Valenciano de Oncología, Valencia, Spain; ^3^ Department of Pathology, Fundación Instituto Valenciano de Oncología, Valencia, Spain; ^4^ Department of Urology, Fundación Instituto Valenciano de Oncología, Valencia, Spain; ^5^ Department of Health Sciences, University of Milano-Bicocca, Monza, Italy

**Keywords:** insulin-like growth factor receptor 1, prostate cancer, *ETS* fusion genes, anti-IGF-1R agents

## Abstract

Identifying patients who may benefit from targeted therapy is an urgent clinical issue in prostate cancer (PCa). We investigated the molecular relationship between *TMPRSS2-ERG* (T2E) fusion gene and insulin-like growth factor receptor (IGF-1R) to optimize the use of IGF-1R inhibitors.

IGF-1R was analyzed in cell lines and in radical prostatectomy specimens in relation to T2E status. ERG binding to *IGF-1R* promoter was evaluated by chromatin immunoprecipitation (ChIP). Sensitivity to anti-IGF-1R agents was evaluated alone or in combination with anti-androgen abiraterone acetate *in vitro* at basal levels or upon ERG modulation.

IGF-1R analysis performed in PCa cells or clinical samples showed that T2E expression correlated with higher IGF-1R expression at mRNA and protein levels. Genetic modulation of ERG directly affected IGF-1R protein levels *in vitro*. ChIP analysis showed that ERG binds *IGF-1R* promoter and that promoter occupancy is higher in T2E-positive cells. IGF-1R inhibition was more effective in cell lines expressing the fusion gene and combination of IGF-1R inhibitors with abiraterone acetate produced synergistic effects in T2E-expressing cells.

Here, we provide the rationale for use of T2E fusion gene to select PCa patients for anti-IGF-1R treatments. The combination of anti-IGF-1R-HAbs with an anti-androgen therapy is strongly advocated for patients expressing T2E.

## INTRODUCTION

Chromosomal translocations are genetic lesions that are produced by illegitimate recombination events between two non-homologous chromosomes or within the same chromosome and that result in chimeric genes [1]. Although fusion genes have been considered exclusive mutations of lymphomas, leukemias and sarcomas, several tumor-specific rearrangements have been recently identified in carcinomas. In particular, in 2005, a chromosomal rearrangement leading to the fusion of the androgen-regulated gene *TMPRSS2* and one of the *ETS* genes, predominantly *ERG*, was described as being expressed in 40–70% of prostate cancers (PCas) from a radical prostatectomy series [2]. PCa is one of the most commonly diagnosed cancers in adult men, accounting for 10% of cancer deaths in Europe [3]. PCa progression is accompanied by genetic mutations, including *TMPRSS2-ERG* (T2E) rearrangement, which is considered an early event because it is found in localized disease more frequently than in high-grade prostatic intraepithelial neoplasia (PIN) [4]. Because *TMPRSS2* contributes only untranslated sequences, the fusion gene results in the overproduction of a truncated ERG protein (tERG) [2, 5]. ERG shares with other ETS transcription factors the same DNA-binding domain that recognizes the 5′-GGAA/T-3′ motif. ETS proteins are considered proto-oncogenes because they control the expression of target genes involved in cell proliferation, apoptosis and invasion [6]. Studies exploring the functional significance of truncated ERG protein are controversial but suggest that ETS activation promotes epithelial-mesenchymal transition (EMT) and invasiveness [5, 7, 8]. Nevertheless, T2E has been reported as insufficient to induce a transformed phenotype but instead to cooperate with other mutations [9]. We analyzed the impact of T2E on the insulin-like growth factor (IGF) system. The IGF system is composed of three receptors [insulin receptor (IR), IGF-1 receptor (IGF-1R) and mannose 6-phosphate receptor (M6P/IGF-2R)], three ligands (insulin, IGF-1, IGF-2), and six known types of circulating IGF-binding proteins (IGFBP1–6) that modulate the bioavailability and bioactivity of the IGFs [10, 11]. The role of the IGF system and particularly IGF-1R in human cancer has been widely documented [11]. In the prostate, IGF-1R plays a critical role in normal gland growth and development, as well as in cancer initiation and progression [12]. Epidemiologic studies have associated circulating IGF-1 levels with risk of developing disease [13–15]. However, numerous experimental and clinical studies have produced controversial evidence, suggesting a need for further studies. Indeed, although the intensity of IGF-1R immunostaining has generally been reported to increase from benign prostatic hyperplasia (BPH) to PIN to carcinoma [16], several studies have not confirmed this linear relationship and have reported that reduced IGF-1R is associated with hyperplasia and proliferation or metastatic lesions [17, 18]. Despite this variation may be due to technical factors, clinical studies evaluating the prognostic role of IGF-1R expression have also provided controversial results, reporting either positive or negative associations between receptor expression levels and patient outcome [19, 20]. In addition, phase II studies using IGF-1R inhibitors have failed to demonstrate efficacy in castration-resistant PCa (CRPC) patients [21, 22], putatively due to incomplete pathway blockade, onset of resistance mechanisms or lack of a suitable patients selection. A better understanding of the molecular determinants of aberrant IGF-1R expression in prostate tumors is thus required to define subgroups of patients who may benefit from anti-IGF-1R therapies. In this study, we demonstrated that T2E directly binds the *IGF-1R* gene promoter, thus affecting its expression and treatment sensitivity in PCa.

## RESULTS

### tERG directly binds to the *IGF-1R* promoter in prostate cells and modulates IGF-1R expression

**Figure 1:**
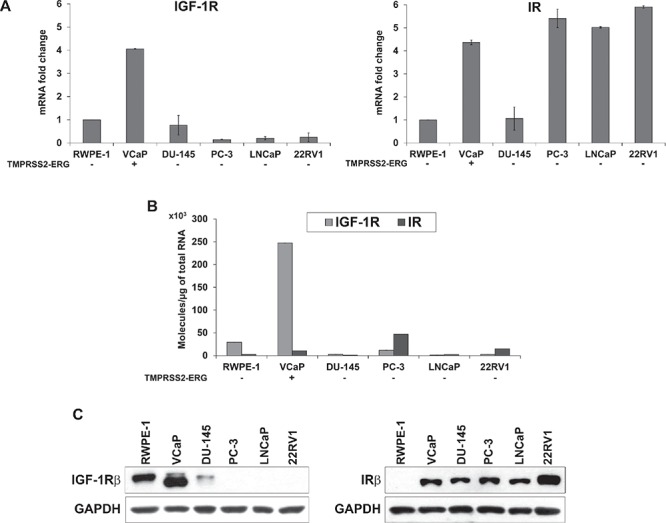
Evaluation of IGF-1R and IR basal expression in prostate cell lines **(A)** Relative mRNA expression levels of *IGF-1R* and *IR* in prostate cancer cell lines. The RWPE-1 cell line was used as a calibrator (2^−ΔΔCt^ = 1). The columns represent the mean values of two independent experiments, and the bars represent the SE. **(B)** Absolute *IGF-1R* and *IR* mRNA quantification was assessed in the panel of cells. **(C)** Protein expression levels of receptors in prostate cells. The blots are representative of two independent experiments.

A panel of five prostate cancer cell lines, VCaP, DU-145, PC-3, LNCaP and 22RV1, characterized by different expression levels of the androgen receptor (AR) and T2E gene fusion, and non-malignant RWPE-1 prostate cells (Supplementary Figure S1) was analyzed for the expression of different components of the IGF system. No IGF-1 or IGF-2 expression was found in the cell lines (data not shown), confirming the paracrine activation of the pathway in this tumor. IR expression is generally higher in PCa cell lines with respect to normal cells (Figure 1). This difference is particularly evident at the protein level and does not appear to reflect a regulation at the transcriptional level. In contrast, IGF-1R expression is generally low in malignant cells, with the only notable exception of VCaP cells, which express the T2E fusion gene. These data were confirmed at the mRNA and protein levels, thus supporting regulation at the transcriptional level for IGF-1R expression (Figure 1).

**Figure 2:**
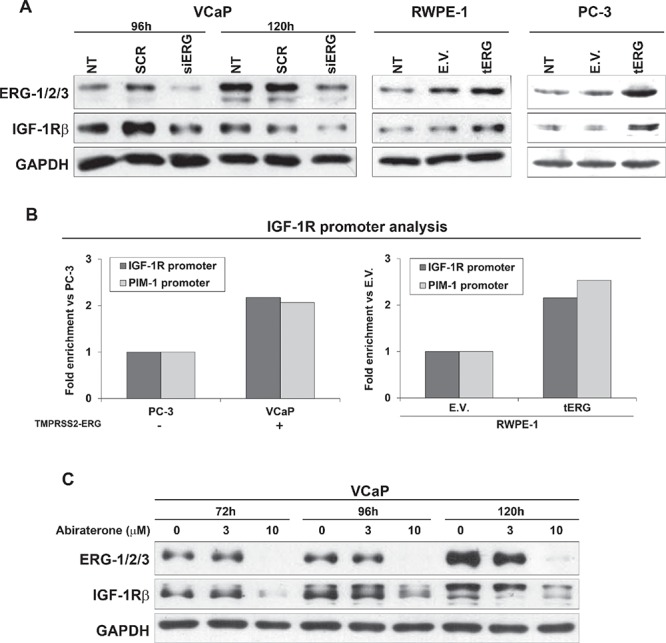
tERG-dependent IGF-1R induction in prostate cancer cells **(A)** siRNA knockdown of ERG (siERG) in VCaP induces a decrease in IGF-1R levels compared with non-treated control (NT) or non-targeting siRNA (SCR) controls, whereas IGF-1R is over-expressed in RWPE-1 and PC-3 cells transfected with tERG compared with empty vector-transfected cells. The blots are representative of two independent experiments. GAPDH is shown as a loading control. **(B)** A ChIP assay was performed on VCaP and PC-3 prostate cancer cells, as well as on tERG- or empty vector-transfected RWPE-1 cells. ERG was precipitated with an anti-ERG-1/2/3 antibody. The results were obtained by quantitative RT-PCR. The data represent the recovery of each DNA fragment relative to the total input DNA. **(C)** Abiraterone acetate treatment induces down-regulation of ERG in VCaP cells and, consequently, down-regulation of IGF-1R. Cells were treated with abiraterone (3 and 10 μM) for the indicated time points. Representative blots are shown. GAPDH was used for normalization.

To better understand the role of tERG in IGF-1R modulation, IGF-1R protein levels were analyzed after ERG siRNA transfection in VCaP cells. A decrease in IGF-1R was evident 96 h and 120 h after silencing. Conversely, IGF-1R protein expression was increased both in the non malignant RWPE-1 and malignant PC-3 cells stably transfected for tERG overexpression (RWPE-1_tERG and PC-3_tERG, respectively; Figure 2A), confirming the correlation between IGF-1R and the fusion gene. Moreover, an anti-ERG chromatin immunoprecipitation (ChIP) assay was performed in VCaP and parental PC-3 cells, which express ERG at high or low levels, respectively, as well as in RWPE-1_tERG cells. ChIP analysis indicated that ERG binds the *IGF-1R* gene promoter, and the amount of binding was higher in cells with tERG expression (Figure 2B). No consensus sequences were present in the promoter of *IR* (data not shown). Because the T2E fusion gene is regulated by androgens, the naturally expressing T2E VCaP cells were treated with abiraterone acetate, and IGF-1R protein levels were investigated upon stimulation. Abiraterone acetate is a second-generation anti-androgen drug that blocks the synthesis of androgens through the inhibition of 17 α-hydroxylase/C17, 20 lyase (CYP17A1). VCaP cells were treated for 72, 96 and 120 h with two concentrations of abiraterone acetate, and western blotting analysis showed that together with a strong ERG down-regulation, IGF-1R levels decreased upon 10 μM treatment in VCaP cells (Figure 2C).

### tERG overexpression increases sensitivity to anti-IGF-1R agents

**Figure 3:**
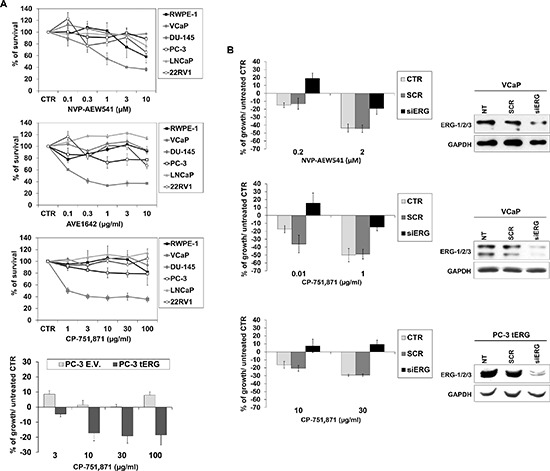
Efficacy of anti-IGF-1R agents in prostate cancer cells **(A)** Cell growth was assessed using an MTT assay after a 72-h exposure to CP-751,871 or AVE1642, two anti-IGF-1R-HAbs, and NVP-AEW541, an anti-IGF-1R tyrosine kinase inhibitor (TKI) in prostate cell lines. PC-3 cells transfected with tERG or PC-3 empty vector-transfected cells were treated with indicated doses of CP-751,871 for 72 h. The results are displayed as the percentage of survival relative to controls. Points, mean of two independent experiments; bars, SE. **(B)** Reversion of sensitivity to anti-IGF-1R therapies by ERG knockdown. ERG silencing was achieved in VCaP or PC-3_tERG cells after a 48 h transfection of siERG (100 nM) or scrambled control siRNA (100 nM); GAPDH was used as a loading control. The transfected cells were treated as described in the Materials and Methods. Cell survival is shown as the percentage of growth respect to untreated control. The data represent the mean values of two independent experiments, and the bars represent the SE.

**Figure 4:**
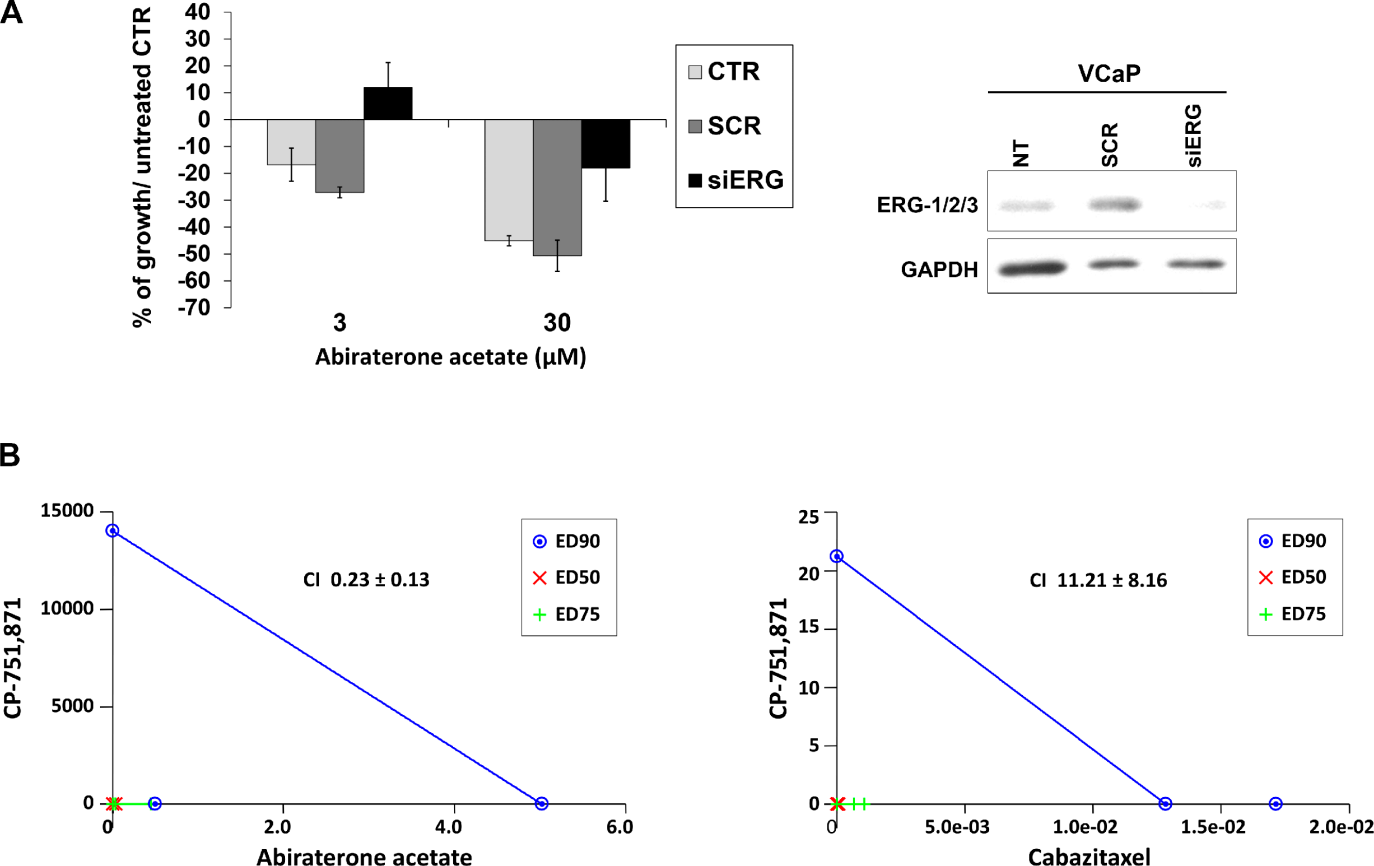
The combination of an IGF-1R inhibitor with an anti-androgen drug results in synergistic effects in TMPRSS2-ERG-positive cells **(A)** ERG was silenced in VCaP cells with siERG (100 nM) or scrambled control siRNA (100 nM); GAPDH was used as a loading control. Cells were treated with abiraterone acetate for 72 h at the indicated doses, and the survival percentage with respect to untreated control is shown. The data represent the mean values of two independent experiments, and the bars represent SE. **(B)** The effects of simultaneous combined treatments of CP-751,871 in association with abiraterone acetate or cabazitaxel. Individual doses of CP-751,871, abiraterone acetate or cabazitaxel to achieve 90% growth inhibition (blue line; ED90), 75% growth inhibition (green line; ED75) and 50% (red line; ED50) growth inhibition are plotted on the x- and y-axes. CI values are represented by the points above on (indicating synergy), or below (indicating antagonism) the lines. The CI values representing ED90 are reported.

PCa cell lines were exposed to increasing concentrations of CP-751,871 or AVE1642, two anti-IGF-1R-HAbs, as well as NVP-AEW541, a selective IGF-1R tyrosine kinase inhibitor (TKI) [23–26]. As shown in Figure 3A, only VCaP cells showed remarkably high sensitivity to all anti-IGF-1R agents compared with the other PCa cell lines. Accordingly, PC-3_tERG cells showed increased sensitivity to CP-751,871 treatment compared to empty vector transfected cells. To address the role of the T2E/IGF-1R axis in influencing sensitivity to IGF-1R inhibitors, VCaP cells as well as PC-3_tERG cell line were deprived of ERG. The level of ERG expression significantly influenced the efficacy of anti-IGF-1R agents because its silencing cells reverted cell sensitivity toward CP-751,871 or NVP-AEW541 (Figure 3B). Notably, in prostate cancer, several clinical trials have investigated the effects of IGF-1R inhibitors in combination with other drugs, such as mitoxantrone (NCT00683475) or docetaxel [27]. Thus, because *TMPRSS2-ERG* expression is driven by androgens, we first investigated the response to abiraterone acetate in VCaP cells upon ERG silencing and observed that these genetically modified cells showed a significant decrease in sensitivity to abiraterone stimulation (Figure 4A). Interestingly, the simultaneous administration of anti-IGF-1R CP-751,871 HAbs and abiraterone but not cabazitaxel, a microtubule inhibitor recently introduced in PCa treatment, induced synergistic antiproliferative effects in VCaP cells (Figure 4B). Conversely, combined treatment of CP-751,871 and abiraterone gave subadditive effects in T2E-null DU-145 and LNCaP cell lines (CI = 2.88 ± 1.17 and CI > 100, respectively).

### IGF-1R levels are associated with T2E expression in clinical samples

**Figure 5:**
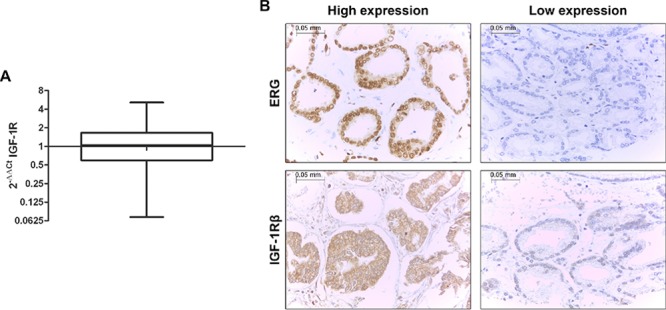
The fusion gene is directly correlated with IGF-1R in prostate cancer patients **(A)** *IGF-1R* expression profile in 270 FFPE primary prostate cancer samples analyzed by qRT-PCR. Normal tissues were used as calibrator. *IGF-1R* was not differentially expressed with respect to normal tissue (median = 1.04; range = 0.07–5.12). **(B)** Representative expression of ERG (top) and IGF-1R (bottom) in prostate cancer tissue array samples by immunohistochemistry (magnification, x40). The cases were classified as ‘high-expressors’ when medium or high positivity was present and ‘low-expressors’ when no staining or low positivity was observed.

To confirm the clinical relevance of our experimental observations, we examined the gene expression levels of *IGF-1R* by qRT-PCR in a retrospective cohort of 270 primary prostate tumors (Figure 5A). Fisher's test revealed an association between *IGF-1R* and T2E expression in clinical samples (*p* = 0.008). In particular, patients harboring the fusion gene showed higher *IGF-1R* mRNA levels, in keeping with the increased binding of ERG to the *IGF-1R* promoter. This association was confirmed at the protein level. We analyzed the protein expression of ERG and IGF-1R in the same series of patients (Table 1). IGF-1R and ERG expression at the mRNA and protein levels (evaluation scores are reported in the Materials and Methods) were significantly correlated (*p* = 0.047 and *p* < 0.0001; Fisher's test, respectively). As observed at the mRNA level, IGF-1R protein expression was also found to be significantly associated with ERG expression (*p* < 0.0001; Fisher's test), further verifying the association between IGF-1R and T2E (Figure 5B).

**Table 1:** Clinicopathologic features of the analyzed series

Parameter	qRT-PCR (*n* = 270)		IHC (*n* = 243)	
	No. Pts	%	No. Pts	%
Age				
≤ 55	15	5, 6	12	5
56–65	81	30	74	31
66–75	138	51, 1	124	52, 1
> 75	36	13, 3	28	11, 7
Gleason-sp:				
2–6	109	40, 4	87	36, 4
7	129	47, 8	123	51, 4
Greater than 7	32	11, 9	29	12, 1
PSA (ng/ml):				
10 or less	154	57	132	55, 6
10–20	74	27, 6	69	29, 1
Greater than 20	40	14, 9	36	15, 1
cT:				
cT2b or less	248	92, 2	219	92
cT3a or greater	21	7, 8	19	7, 9
pT:				
pT2 or less	135	50	115	48, 1
pT3 or greater	135	50	124	51, 8
pN*:				
pN0	236	95, 2	209	95, 4
pN1 or greater	12	4, 8	10	4, 5
Margins:				
Negative	137	50, 7	116	48, 5
Positive	133	49, 3	123	51, 4
TMPRSS2-ERG**				
Negative	92	34, 1	102	46, 5
Positive	178	65, 9	117	53, 4
IGF-1R***				
Low expressors	82	30, 4	55	24, 7
High expressors	188	69, 6	167	75, 2

SP, specimen; cT, clinical stage; PSA, prostatic specific antigen; pN, lymphnode pathological stage

* Lymphadenectomy was limited to the obturator fossa in most of the cases at the inclusion period

** IHC ERG expression was not detectable in 24/243 and negative in 85/219 cases (39%)

*** IHC IGF-1R expression was not detectable in 21/243 cases and negative in 12/222 of the samples (5%).

## DISCUSSION

The T2E fusion gene constitutes a critical event in development of PCa [2, 28], but other genetic alterations, such as loss of PTEN and PI3K pathway activation, are also required to induce malignant transformation [8]. Here, we provide evidence that IGF-1R is a target of tERG from T2E translocation and that this interaction has important implications in the field of personalized treatment through biomarker-driven patient selection. ChIP analysis showed ERG binding to the *IGF-1R* gene promoter, suggesting a direct transcriptional regulation of *IGF-1R* by ERG. Furthermore, we found greater ERG recruitment to the *IGF-1R* promoter in VCaP cells compared with PC-3 cells expressing low ERG levels, as well as in RWPE-1 cells that over-expressed tERG compared with the empty vector. As a proof of concept, androgen deprivation induced by abiraterone acetate treatment in the androgen-responsive VCaP cells caused a significant decrease in ERG expression, as previously reported [29], but also a consequent inhibition of IGF-1R, confirming the presence of a T2E/IGF-1R androgen-regulated axis. Considering that RWPE-1 represents a model of non-tumorigenic immortalized cells but that VCaP cells are representative of advanced disease, the data indicate that the T2E/IGF-1R axis may represent a constant mechanism along different stages of pathology with putatively different consequences depending on pathological stage. The relationship between T2E and IGF-1R was also confirmed in radical prostatectomy specimens; patients expressing the fusion gene exhibited higher IGF-1R expression.

The *IGF-1R* gene has been identified as a molecular target for a number of stimulatory transcription factors and inhibitory proteins with important implications in cancer [30]. Aberrant fusion products, such as EWS-WT1 or EWS-FLI, the genetic hallmarks of desmoplastic small round cell tumor or Ewing sarcoma, were found to act as transactivators for the IGF-1R system, providing a selective growth advantage to tumor cells [31, 32]. From a biological standpoint, the T2E/IGF-1R axis may be assumed to participate in establishing a biologically distinguished cellular context and promote a malignant cellular phenotype compared with cells that do not express T2E. From the clinical standpoint, this mechanism provides the rationale for the selective use of anti-IGF-1R agents for patients expressing the fusion gene. The contribution of IGF-1R to prostate carcinogenesis and progression remains controversial, but epidemiological, preclinical and clinical results indicate that IGF-1R overexpression plays an important role in the pathogenesis of CRPC [33]. This evidence in particular led to the enrollment of CRPC patients in several clinical trials investigating the effects of IGF-1R inhibitors. However, these clinical trials verified only very modest clinical benefits from IGF-1R inhibition [22, 27] and resulted in discontinuing the development of most of anti-IGF-1R agents. Here, we demonstrated that only PCa cells that express the translocation and therefore have higher IGF-1R expression displayed potentially interesting sensitivity to anti-IGF-1R agents. Accordingly, ERG silencing caused a decrease in treatment sensitivity, thus supporting the idea that only patients with PCa presenting with T2E may benefit from anti-IGF-1R therapy. This idea is in line with previous evidence demonstrating how PARP1 inhibitors blocked ETS-positive but not ETS-negative prostate cancer xenograft growth [34]. In addition, consistent with the observation that T2E-positive CRPC tumors display a better response to anti-androgen treatment compared with T2E-negative tumors [35], we found that sensitivity to abiraterone acetate significantly decreased upon ERG silencing. Abiraterone acetate is a selective small molecule inhibitor of CYP17, an enzyme that catalyzes generation of androgens and estrogens. In the clinic, the onset of androgen receptor-linked resistance mechanisms in CRPC patients treated with abiraterone is an important limitation, and the identification of a druggable target involved in the androgen receptor pathway may be an interesting opportunity to overcome resistance [36]. Combined therapies with abiraterone and targeted agents, such as Src inhibitors [37] or PI3K pathway inhibitors [38], have been proposed. Our results provide evidence for the first time of a beneficial combination of abiraterone acetate and anti-IGF-1R agents. In VCaP cells, the association of anti-IGF-1R CP-751,871 HAb with abiraterone acetate produced synergistic effects, supporting the idea that the concurrent use of the two targeted agents deprive tERG-expressing cells of fundamental signaling pathways that operate in concert to sustain cell proliferation.

Overall, we suggest the application of T2E as a biomarker for patient selection in the field of personalized medicine. We demonstrated that IGF-1R is an important target of tERG and that this interaction leads to a higher IGF-1R expression in cell lines and patients. Thus, we observed a good response to IGF-1R inhibition in T2E-positive cells compared with T2E-negative cells. Considering that such a mechanism is driven by androgens, we provide the rationale for combining anti-IGF-1R agents to anti-androgen therapy in the subpopulation of patients expressing T2E.

## MATERIALS AND METHODS

### Cell lines

Prostate cancer cell lines PC-3, LNCaP, DU-145, VCaP were obtained from the American Type Culture Collection (ATCC). 22RV1 prostate cancer cell line was purchased from Sigma Aldrich. Immortalized non-malignant prostate cell line RWPE-1 and stable trasfectants RWPE-1_tERG or RWPE-1_empty vector were kindly provided by Dr. Gambacorti-Passerini, University of Milano-Bicocca [39]. PC-3, LNCaP and DU-145 cells were cultured in Iscove's Modified Dulbecco's Medium (IMDM) (Lonza). RWPE-1 and transfectant cells were maintained in keratinocyte-serum free medium supplemented with epidermal growth factor and bovine pituitary extract (Life Technologies Inc.). 22RV1 cells were maintained in RPMI 1640 (Gibco) while VCaP cells were maintained in Dulbecco's Modified Eagle's Medium (DMEM) (Sigma) implemented with L-glucose and bicarbonate. IMDM, RPMI and DMEM media were supplemented with 10% inactivated Fetal Bovine Serum (FBS) (Lonza) and 100 units/ml penicillin and 100 μg/ml streptomycin. Cells were maintained at 37°C in a humidified 5% CO_2_ atmosphere. All cell lines were tested for mycoplasma contamination every 3 months by MycoAlert mycoplasma detection kit (Lonza) and were recently authenticated by STR PCR analysis using genRESVR MPX-2 and genRESVR MPX-3 kits (Serac). The following locus were verified: D3S1358, D19S433, D2S1338, D22S1045, D16S539, D18S51, D1S1656, D10S1248, D2S441, TH01, VWA, D21S11, D8S1179, FGA, SE33.

### Clinical prostate specimens

Formalin fixed and paraffin-embedded (FFPE) blocks corresponding to PCa patients were retrieved from the archives of the Biobank of the *Fundación Instituto Valenciano de Oncología* according to the following criteria: specimens obtained from radical retropubic prostatectomies from 1996 to 2002 and no history of previous treatment for PCa (including androgen deprivation therapy or chemotherapy prior to surgery). We identified 270 cases that met these criteria. All patients gave written informed consent for tissue donation for research purposes before tissue collection, and the study was approved by FIVO's Institutional Ethical Committee (ref. number. 2010-19). Clinical data were reviewed from clinical records and stored in a PCa-specific database. Patient characteristics, including the T2E fusion gene status, and demographics are shown in Table 1. Combined Gleason score was uniformly regarded by the same uro-pathologist (AC). For comparative and calibration purposes, we also analyzed 10 samples of normal prostate tissue obtained from patients operated of radical cystectomies without pathological evidence of prostatic disease. T2E gene fusion status was determined by RT-PCR and fluorescent *in situ* hybridization (FISH) as already described [40] and quantitative RT-PCR.

### Gene expression analysis

Cell lines total RNA (2 mg) was extracted with TRIzol (Invitrogen) and purified by precipitation with isopropanol. Oligo dT primers (Applied Biosystems) were used to reverse transcribe RNA. Isolation of RNA from paraffin-embedded tissue was performed using RecoverAll™ Total Nucleic Acid Isolation Kit (Ambion) following providers' specifications and reverse transcription was performed with High Capacity cDNA Reverse Transcription Kit (Applied Biosystems) according to manufacturer's indications. For cell line analysis, Quantitative Real-Time PCR was performed on ABI Prism 7900 (Applied Biosystems) using TaqMan (*IGF-1R*) or SYBR Green assays (*IR*) (Applied Biosystems) as previously reported [26]. Primer Express software (Applied Biosystems) was used to design appropriate primer pairs for reference gene (glyceraldehyde-3-phosphate dehydrogenase) [26]. Clinical samples were analyzed using ABI 7500-Fast Thermocycler Sequence Detection System (Applied Biosystems), according to manufacturer's instructions. Predesigned TaqMan probes for target genes *IGF-1R* (Hs00181385_m1), T2E (Hs03063375_ft) as well as for endogenous control *β-2-microglobulin* (Hs99999907_m1) were used (Applied Biosystem). Two replicates per gene were considered. Relative quantification analysis was performed on ΔΔCt method [41]. cDNA from normal human prostate samples was used as calibrator for comparative analysis of PCa cases. Absolute quantification assay was performed for the measurement of total *IR* and *IGF-1R* [42].

### Western blotting

Cell lysates were prepared and processed as previously described [43]. Membranes were incubated overnight with the following primary antibodies: anti-IGF-1Rβ, anti-IRβ, anti-GAPDH, anti-LAMIN B, anti-ERG-1/2/3 (Santa Cruz Biotechnology); anti-AR (Cell Signaling Technology); anti-rabbit or anti-mouse antibodies conjugated to horseradish peroxidase (GE Healthcare) were used as secondary antibodies.

### Drugs

Anti-IGF-1R drugs were kindly provided by: ImmunoGen Inc. (AVE1642, a humanized version of anti-IGF-1R EM164 antibody), Pfizer (CP-751,871/Figitumumab), and Novartis (NVP-AEW541). Abiraterone acetate (S1123) and Cabazitaxel (S3022) were purchased by Selleckchem.

### *In vitro* assays

To assess drug sensitivity, MTT assay (Roche) was used according to manufacturer's instructions. Cells were plated into 96 well-plates (10, 000 cells/well). After 24 hours, various concentrations of AVE1642 (0.01–50 μg/ml), NVP-AEW541(0.03–5 μM), Figitumumab (0.5–500 μg/ml) were added and cells exposed to these drugs for up to 72 hours. phCMV2_HA_tERG plasmid containing the cDNA of the translated sequence of *TMPRSS2-ERG* (isoform 9) and phCMV2 empty vector were kindly provided by Dr. Gambacorti-Passerini, University of Milano-Bicocca [39]. PC-3 cell line was stably transfected with Calcium Phosphate Transfection Kit (Invitrogen) accordingly to manufacturer's instruction and selected for geneticin (Sigma) resistance at 0.75 mg/ml. PC-3_tERG and PC-3 empty vector transfected cells were treated with CP-751,871 (3, 10, 30, 100 μg/ml) for up to 72 hours and sensitivity was assessed with Trypan Blue cell count. Short interfering RNA knockdown of ERG was performed with siRNA from Thermo Scientific Dharmacon: siGENOME_siRNA (D-003886-01) as reported in Tomlins et al. [5] and Magistroni et al. [39]. siGENOME_non targeting_siRNA was used as control (D-001210-01-05). siRNA was transfected in VCaP or PC-3_tERG cells using siport NeoFX transfection agent (Life Technologies Inc.) according to manufacturer's instructions. Silencing was assessed after 48, 72, 96 and 120 hours from transfection. VCaP cells were pre-treated with ERG siRNA (100 nM) for 48 hours and then exposed to CP-751,871 (0.01–1 μg/ml), NVP-AEW541 (0.2–2 μM) or Abiraterone (3–30 μM) for 72 hours. PC-3_tERG cells were pre-treated with ERG siRNA (100 nM) for 48 hours and then exposed to CP-751,871 (10–30 μg/ml). ERG and IGF-1R protein expression was investigated upon 72, 96 and 120 hours of Abiraterone treatment (3–10 μM). For combined treatments, LNCaP, DU-145 and VCaP cells were treated for 72 hours with varying concentrations of CP-751,871 (1–100 μM) and Abiraterone (1–100 μM) or Cabazitaxel (0.003–0.3 μM).

### Chromatin immunoprecipitation (ChIP)

ChIP assay was performed as previously described [43, 44] using anti-ERG-1/2/3 antibody (C-17, Santa Cruz Biotechnology). *IGF-1R* promoter was evaluated by Real-Time PCR using the following custom TaqMan assay: forward 5′-AGGAGGAGGAGGAGG AGGAG-3′, reverse 5′-GCAGTTCGCAAGATCGCC-3′ and probe 5′-TTGACTCCGCGTTTCTGCCCCTCG-3′. For the TaqMan assay design TFSEARCH - Searching Transcription Factor Binding Sites, version 1.3 free website was used for the prediction of ETS binding sites in the promoter of *IGF-1R* gene and the sequence spanning from 1041bp to 1051bp was identified as the best. Beacon Designer 4 software was used for the design of the assay spanning from 1005bp to 1114bp. PIM-1 promoter fragment containing ETS consensus sequence was used as immunoprecipitation positive control [39] by Real-Time PCR using the following SYBR Green assay: forward 5′-GTGCTAGGCGAGTGGGAACAACTG-3′ and reverse 5′-AATGACCCAAATTCACCTCCTGAG-3′. Quantification analysis was calculated with the following formula: % of recruitment = 2^ΔCt^ × input chromatin percentage where ΔCt = Ct (INPUT) - Ct (IP:ERG) [45].

### Immunohistochemistry

PCa specimens were incorporated in 11 tissue microarrays (TMA). Two or three representative areas (1 mm in diameter) of each tumor were selected for TMA production by first examining hematoxylin and eosin-stained prostatectomy tumor slides and then sampling tissue from the corresponding paraffin blocks. A tissue microarray instrument (Beecher Instruments) was used for TMA assembly. From TMA blocks, 3-μm-thick sections were immunostained using rabbit anti-human IGF-1Rβ (Santa Cruz Biotechnology) or anti-human ERG clone EP111 polyclonal-Ab (Dako). Percentage of IGF-1R-positive cells and cytoplasmic staining intensity were scored semiquantitatively, forming four groups (from 0 to 3). Cases were scored as low expression when staining intensity was between 0 and 1, and high expression when intensity was 2 and 3.

### Statistical analysis

Differences among means where analyzed using two-sided Student's *t* test. To define drug-drug interactions combination index (CI) was calculated with the isobologram equation [46] using CalcuSyn software (Biosoft). Correlations analysis was performed using Fisher's exact test.

## SUPPLEMENTARY FIGURE


