# Supplementary material for: ERG deregulation induces IGF-1R expression in prostate cancer cells and affects sensitivity to anti-IGF-1R agents
Source: Oncotarget. 2015 Mar 27;6(18):16611–22. doi: 10.18632/oncotarget.3425 (PMC4599293; doi:10.18632/oncotarget.3425)
Supplement: Supplementary file 1 [file oncotarget-06-16611-s001.pdf]

SUPPLEMENTARY FIGURE

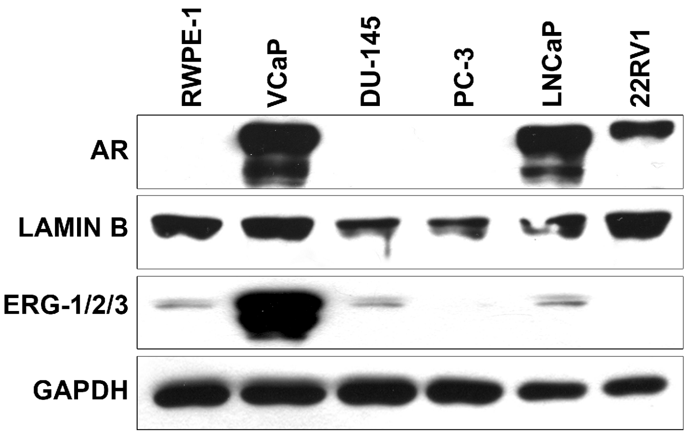

Supplementary Figure S1: Protein expression levels of androgen receptor (AR) and ERG-1/2/3 in prostate cell lines. Blots are representative of two independent experiments. LAMIN B or GAPDH were used as normalization.
